# The Limbic-Prefrontal Network Modulated by Electroacupuncture at CV4 and CV12

**DOI:** 10.1155/2012/515893

**Published:** 2012-01-16

**Authors:** Jiliang Fang, Xiaoling Wang, Hesheng Liu, Yin Wang, Kehua Zhou, Yang Hong, Jun Liu, Lei Wang, Chao Xue, Ming Song, Baoyan Liu, Bing Zhu

**Affiliations:** ^1^Functional Brain Imaging Lab, Department of Radiology, Guang An Men Hospital, China Academy of Chinese Medical Sciences, Beijing 100053, China; ^2^Athinoula A. Martinos Center for Biomedical Imaging, Massachusetts General Hospital, Harvard Medical School, Charlestown, MA 02129, USA; ^3^Department of Acupuncture and Moxibustion, Guang An Men Hospital, China Academy of Chinese Medical Sciences, Beijing 100053, China; ^4^Department of Physical Therapy, Daemen College, Amherst, NY 14226, USA; ^5^Institute of Automation, Chinese Academy of Sciences, Beijng 100190, China; ^6^China Academy of Chinese Medical Sciences, Beijing 100700, China; ^7^Institute of Acupuncture and Moxibustion, China Academy of Chinese Medical Sciences, Beijing 100700, China

## Abstract

fMRI studies showed that acupuncture could induce hemodynamic changes in brain networks. Many of these studies focused on whether specific acupoints could activate specific brain regions and were often limited to manual acupuncture at acupoints on the limbs. In this fMRI study, we investigated acupuncture's modulation effects on brain functional networks by electroacupuncture (EA) at acupoints on the midline of abdomen. Acupoints Guanyuan (CV4) and Zhongwan (CV12) were stimulated in 21 healthy volunteers. The needling sensations, brain activation, and functional connectivity were studied. We found that the limbic-prefrontal functional network was deactivated by EA at CV4 and CV12. More importantly, the local functional connectivity was significantly changed during EA stimulation, and the change persisted during the period after the stimulation. Although minor differences existed, both acupoints similarly modulated the limbic-prefrontal functional network, which is overlapped with the functional circuits associated with emotional and cognitive regulation.

## 1. Introduction

Acupuncture, an ancient medicine practiced in China for thousands of years, has been becoming more and more popular in the modern world; however, its therapeutic mechanism is yet to be fully elucidated [1–4]. During the past two decades, various fMRI studies showed that acupuncture could induce hemodynamic changes in brain functional networks [5–15]. Among these studies, many focused on the specific effects of specific acupoints. Researchers found that visual, auditory, or language-related acupoints elicited fMRI signal responses in their corresponding cortices [6, 16–19]. However, the reproducibility is a subject of debate [20, 21]. The BOLD signal responses were also observed in visual and auditory cortices when stimulating some acupoints that are not related to vision or audition according to the traditional theory [11, 12, 20–29]. Therefore, the hypothetic specific pathway connecting “acupoint-brain-organ” has recently been replaced by the “hypothalamus-pituitary-adrenal axis” hypothesis [30, 31].

Although brain responses vary slightly across different acupoints, researchers found similar central effects. fMRI studies of acupuncture at Hegu (LI4), Zusanli (ST36), and Taichong (LV3) suggested that acupuncture may exert its therapeutic effects via regulation and integration of multiple brain functions that mainly involve the limbic system [5, 7, 8, 11–13, 15, 22, 25, 26, 29]. Our previous studies of acupuncture at Taichong (LV3), Xingjian (LV2), and Neiting (ST44) were in accordance with this finding, and we further observed that acupuncture produced extensive hemodynamic response within the limbic-paralimbic-neocortical network [12].

Nevertheless, as an important doctrine in the acupuncture theory, acupoint specificity is still a strong belief among many acupuncturists. In this study, we aim to investigate the brain response specificity by employing electroacupuncture at two acupoints Guang yuan (CV4) and Zhong wan (CV12) on the abdomen, that were underexplored in previous studies that usually focused on acupoints on the limbs although abdominal acupoints are commonly used in current clinical practice. We have chosen the electroacupuncture because EA has the advantage of unified stimulating parameters compared to manual acupuncture.

Functional connectivity measured by resting-state fMRI has recently emerged as a powerful approach to study brain networks. Resting-state functional connectivity is defined as the temporal correlation of a neurophysiological index measured in different brain areas [31, 32]. The default network, the dominant functional network during the resting state, plays an important role in attention, memory, consciousness, and self-referential processes [33, 34]. In our previous study, it was observed that the acupuncture may modulate the limbic-paralimbic-neocortical network which largely overlaps with the default network [12, 15]. Acupuncture information processing in the brain may involve interactions among specific networks including default network [13–15]. More recently, researchers estimated the degree of local connectivity as well as the degree to which cortical areas serve as “hubs” connecting distributed pathways. It was found that early sensory and motor areas displayed local (modular) connectivity, while connectivity hubs included prefrontal, parietal, and temporal association cortices [35, 36]. These new methods for network analysis will provide a powerful approach to study the central effects of acupuncture.

We hypothesize that (1) electroacupuncture, with its extensive effects on the limbic-paralimbic-neocortical system, will modulate the degree of connectivity in the default network and (2) the effects will persist in the resting state following electroacupuncture.

## 2. Material and Methods

### 2.1. Subjects

Twenty one young healthy volunteers (22 to 28 years old, right-handed, 10 males) were recruited through advertisements. All participants were free of major medical illnesses, history of head trauma, or use of medications within three days prior to the experiments. All participants had no contraindications for MRI scanning. The research protocol was approved by the ethics committee of Guang An Men Hospital and the signed informed consent was obtained from every participant.

### 2.2. Research Protocol

#### 2.2.1. Acupuncture Procedures and Needling Sensation Recording

**Figure 1:**
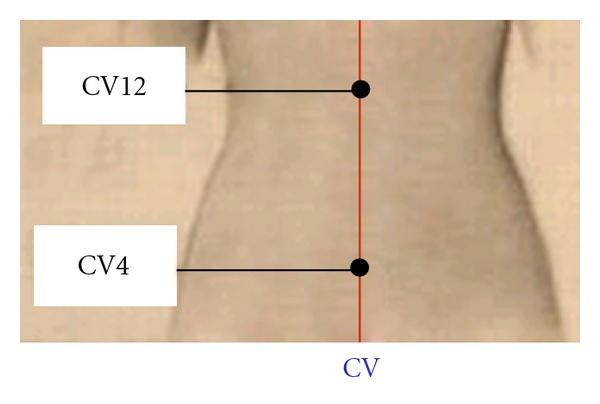
Location of Guanyuan (CV4) and Zhongwan (CV12) at the Conception Vessel.

Acupoints CV4 and CV12 were localized according to Names and Localizations of Acupoints (GB/T12346-2006) (Figure 1) [37]. Silver needles (diameter 0.35 mm, length 40 mm, Huatuo, manufactured by Suzhou Medical Appliance Manufactory, Jiangsu, China) and Han's Acupoint Nerve Stimulator (LH204H, Neuroscience Research Center, Peking University, Beijing, China) were used in this study. Constant bidirectional square current with a frequency of 15 Hz was used for electric stimulation. In order to rule out the possible bias caused by the order of acupuncture procedures, CV4 test was followed by CV12 test with a 10 minute interval in half of the subjects, and CV12 test was followed by CV4 in the second half of the subjects. The participants were in the supine position in the MRI scanner and were unable to see the acupuncture procedures. Prior to the study, acupuncturists instructed participants of possible deqi sensations and asked them to report deqi sensations as well as sharp pain once they felt it. Before needle insertion, the participants were told to rest in the scanner. One electrode of the electric stimulator was attached to the left thenar skin near the thumb. The needle was inserted perpendicularly to the acupoints with a depth of about 2.5 cm. Two acupuncturists managed manual acupuncture using similar moderate reinforcing and reducing method and the participant would report his/her deqi sensation. Another electrode of the stimulator was then attached to the needle handle. Electric current was adjusted to the participant's best tolerated magnitude without noxious (sharp) pain before the imaging acquisition.

**Figure 2:**
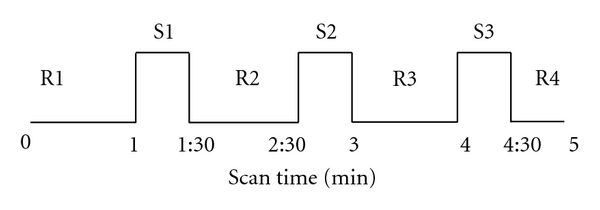
Experimental paradigm (block design): each acupuncture procedure lasted for five minutes, while MR images were acquired. Needle was inserted in the acupoint before the fMRI scan started. R1, 2, 3, and 4 indicate the blocks in which no electric current was delivered; S1, 2, and 3 indicate the electric stimulating blocks.

For each acupoint, the imaging procedure includes four sessions: a resting state session, a sham EA session with needle in place (needle retention without electric stimulation), EA, and a resting state session after EA (after needle removal). Each session was 5 minutes. The experimental paradigm for the EA session was shown in Figure 2. 30-second epochs of electric current delivery (S1, S2, and S3) were interleaved with epochs of rest (R). The needle remained in place without electric stimulation during the rest periods (R1, R2 and R3 are 1 min each, and R4 is 30 sec). After needle removal (end of R4), the participant finished a questionnaire reporting his/her sensations during the EA including soreness, fullness, numbness, warmness, heaviness, coolness, tingling, pressure, dull pain, sharp pain, or any other sensations occurred during the whole EA process. The sensation was rated on the scale of 0–10 (1–3 mild, 4–6 moderate, 7–9 strong, 10 unbearable) [38].

#### 2.2.2. MRI Method

Brain imaging was conducted on a 1.5 Tesla GE Signa MRI system equipped with the standard two channels' Birdcage head coil. T1-weighted high-resolution structural images were acquired with the 3D-FSPGR sequence (matrix 192 × 256, FOV 200 mm, flip angle 15°, slice thickness 1.4 mm). T2-weighted functional images encompassing the whole brain were acquired with the gradient echo EPI sequence (TE 30 ms, TR 2500 ms, matrix 64 × 64, FOV 240 mm, flip angle 90°, slice thickness 3.0 mm, gap 0.5 mm, 41 slices, paralleled by AC-PC line). Image collection was preceded by 4 dummy scans to allow for equilibration of the MRI signal. fMRI scans were performed in the following order: a resting state session prior to the EA, a sham EA session with needle in place (needle retention without electric stimulation), EA, and a resting state session after EA (after needle removal). Each functional scan lasted for 5 minutes with a total of 120 time points.

#### 2.2.3. Statistical Analysis for Needling Sensations

The needling sensation was processed using the SPSS software package (Version11.5). Two sample-paired *t*-tests were performed to compare the intensity of each individual sensation and best tolerated electric current between CV4 and CV12 (thresholded at *P* < 0.05). Chi-square tests and Fischer's exact tests were used to compare the frequencies of sensation components for the paired acupoints. Sharp pain occurred in three subjects during EA at CV12 and in one subject during EA at CV4. Since sharp pain has distinct hemodynamic responses [22, 25], we excluded the data of these four subjects. Therefore, we had 17 subjects for the analysis of sensations. For the fMRI analysis, 18 subjects were included for the analysis of EA at of CV12 and 20 subjects were included for the analysis of EA at CV4.

#### 2.2.4. fMRI Data Analysis

**(1) General Linear Model (GLM) Method**

Imaging preprocessing and statistical analysis were performed using the SPM2 software (Wellcome Department of Cognitive Neurology, London, UK). Data preprocessing included motion correction, normalization to the Montreal Neurological Institute (MNI) stereotactic space, and spatial smoothing with an 8 mm Gaussian kernel. For each EA session, the contrast between electric stimulation on and off was derived with a general linear model. Group analysis was performed using the random-effects model. The *t*-test was performed across all subjects to compare the hemodynamic response between CV4 and CV12. The threshold was set to *P* = 0.05, corrected with 5 contiguous voxels.

**(2) Functional Connectivity Analysis**

MRI analysis procedures were optimized for fcMRI analysis extended from the approach developed by Biswal et al. [33].

In fcMRI analyses, brain voxels can be treated as nodes in a graph, and positive correlations between voxel time series above certain strength can be considered as links or edges between these nodes. This approach allows for the graph-theoretic characterization of fcMRI, for example, using degrees to quantify the number of links or edges connected to a node [35, 36].

In the present study, a computationally efficient approach was used to map the degree of functional connectivity across the brain at the voxel level taking into account topographical neighborhood information for the local and distant distinction [35]. For these analyses, the time course of each voxel from the participant's brain defined within a whole-brain mask was correlated to the time course of every other voxel. Pearson correlation coefficients (*r*) were obtained between voxels. We computed the local degree map by counting for each voxel the number of voxels above a correlation threshold of 0.25 (*r* > 0.25) inside its neighborhood and for the distant degree map by counting for each voxel the number of voxels above the same threshold but outside the neighborhood. In the present study, we chose a sphere of 12 mm radius (approximately 3 voxels around target voxels) as the neighborhood. The degree connectivity map was then standardized by Fisher's *Z* transformation so that maps across participants could be averaged and compared. The *Z* score transformation was computed separately for the local and distant degree connectivity maps. The local and distant degree connectivity was calculated for the resting state, sham EA, EA, and post-EA sessions. The maps were then compared between CV4 and CV12.

## 3. Results

### 3.1. Psychophysical Results: Comparison of Needling Sensations between Acupoints

**Figure 3:**
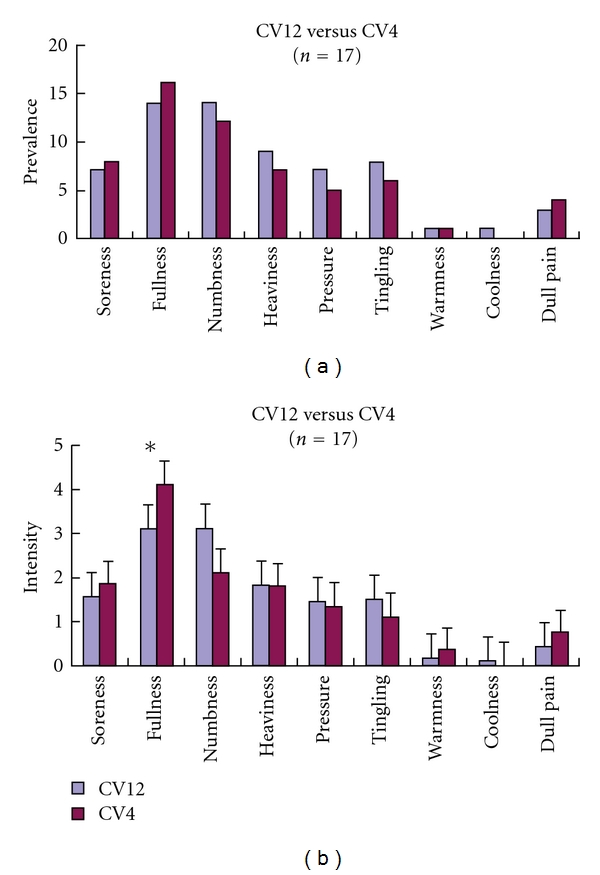
(a) The comparison of sensation prevalence between EA at CV4 and EA at CV12. No significant difference (*P* > 0.05) was observed. (b) The comparison of EA sensation intensity between CV4 and CV12 fullness was stronger in CV4 than CV12 (*P* = 0.02).

Fullness sensation was stronger for CV4 than CV12 (*P* = 0.02). However, no significant difference was found in the prevalence and intensity of other sensations or the amplitude of electric current between these two acupoints (3 mA ± 1.06 for CV4, 3.12 mA ± 0.78 for CV12, *P* = 0.61). The sensations associated with EA at CV4 were fullness, numbness, soreness, and heaviness in descending order for intensity and prevalence, whereas at CV12, the sensations were fullness, numbness, heaviness, and tingling. Dull pain, warmness, and coolness rarely occurred or occurred but in a low intensity for both acupoints (Figures 3(a) and 3(b)).

### 3.2. Hemodynamic Responses

#### 3.2.1. Networks Activated and Deactivated by EA at CV4 and CV12

**Figure 4:**
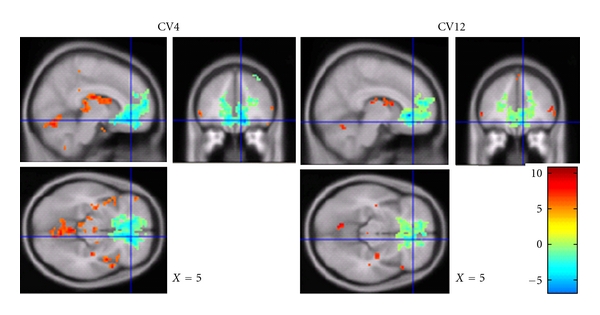
Brain activation and deactivation during EA at CV4 and CV12. Group analysis CV4 (*n* = 20) versus CV12 (*n* = 18) (*P* = 0.05, corrected 5 voxel, MNI coordinates. Strong and prevalent deactivation in ventral medial prefrontal cortex and anterior cingulate cortex was observed for both conditions. There was no significant difference between two acupoints.

For both acupoints, the limbic-prefrontal network including the ventral medial prefrontal cortex (BA10), anteroinferior portion of the anterior cingulate cortex (BA24/32, BA25) were significantly deactivated. The somatosensory regions and its associated regions-somatosensory region II, thalamus, insula, supplementary motor area (BA6), vermis of the cerebellum (III, IV), cortex of the cerebellar hemispheres, and paraaqueductal gray (PAG) were similarly activated for both acupoints (Figure 4).

 No significant difference in activation or deactivation was observed between the acupoints CV4 and CV12 (random-effects model, *P* ≤ 0.05 corrected 5 voxels).

#### 3.2.2. Analysis of Functional Connectivity during EA

**(1) Local and Distant Connectivity during Resting State, Sham EA, and EA Stimulation**

During the resting state prior to acupuncture procedures, the hubs of local and distant connectivity were similar to the hubs as reported in previous studies [35, 36].

**Figure 5:**
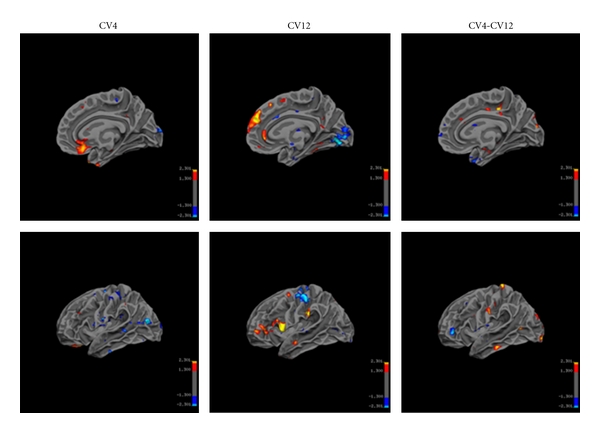
Sham EA at CV4 and CV12 caused moderate changes of local FC. Local connectivity in subgenual of anterior cingulate cortex was bilaterally enhanced for CV4. Local connectivity was enhanced in the left medial middle frontal cortex and pregenual of the anterior cingulate cortex, the right insular and operculum for sham EA at CV12. Local connectivity decreased in bilateral visual cortex and somatosensory cortex during sham EA at CV12. No significant difference was found between two acupoints. (for CV4, *n* = 20, for CV12, *n* = 18, *r* > 0.25, *P* < 0.05).

To test if needle retention (sham EA) causes changes in network property, local and distant functional connectivity was measured in the sham condition for both acupoints. In comparison with the resting state, sham stimulation at each acupoint only induced moderate changes in local functional connectivity (Figure 5) but not in the distant connectivity. The local connectivity in subgenual of anterior cingulate cortex was bilaterally enhanced when the needle was retained at CV4 without electrical stimulation. For the sham condition of CV12, left medial middle frontal cortex and pregenual of the anterior cingulate cortex, the right insular and operculum showed enhanced local connectivity. Decreased local connectivity in the bilateral visual cortex and somatosensory cortex was observed during the sham condition of CV12. However, no significant difference was found between these two acupoints.

**Figure 6:**
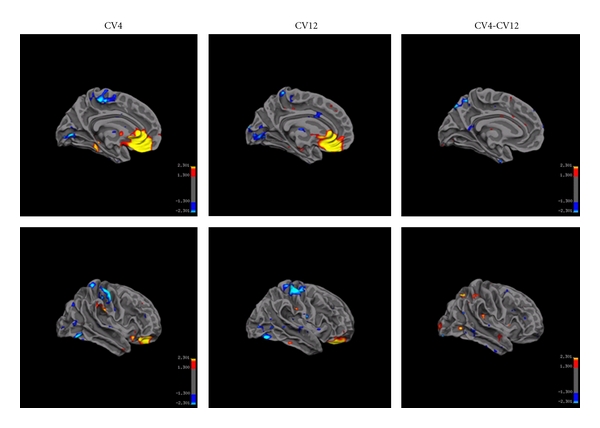
EA at CV4 and CV12 caused similar enhancement of local connectivity in ventral medial prefrontal cortex and anterior cingulate cortex and the decreased local connectivity in primary somatosensory cortex and visual cortex. No significant difference was found between two acupoints. (for CV4, *n* = 20, for CV12, *n* = 18, *r* > 0.25, *P* < 0.05).

In comparison with the resting state, EA stimulation at each acupoint induced significant changes in local functional connectivity (Figure 6). The differences were mainly characterized by the apparently enhanced local connectivity of the limbic-prefrontal network (medial portion of the inferior frontal lobe and anteroinferior portion of the anterior cingulate cortex). Orbital gyrus, hippocampus, and parahippocampus that are among the limbic-paralimbic-neocortical structures also showed some increase in local connectivity. The local connectivity was significantly reduced in the somatosensory, motor, supplementary motor areas, and primary visual cortex. For local connectivity, although similarity was dominant for EA at CV4 and CV12, sporadic differences did exist. Local connectivity was slightly enhanced in the inferior portion of lateral parietal lobe, visual cortex, and lateral portion of the temporal lobe during EA at CV4, whereas enhancement of connectivity was more prominent in the anterior portion of the middle cingulate cortex and the primary somatosensory area with EA at CV12. For distant connectivity, no significant difference was found between these two acupoints.

**(2) Post-EA Effects for CV4 and CV12**

**Figure 7:**
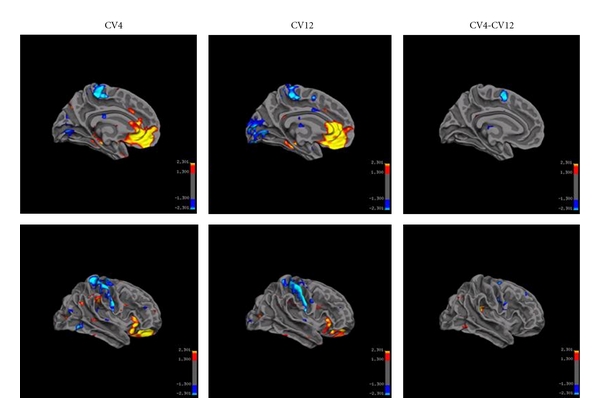
The post-EA effects (local functional connectivity). Local connectivity was enhanced (yellow and red) in medial frontal cortex, orbital gyrus, and anteroinferior portion of the anterior cingulate cortex, followed by hippocampus, parahippocampus, substantia nigra in the mid brain, and superolateral gyrus of the frontal lobe. Decreased local functional connectivity (blue) was found in primary somatosensory cortex, visual cortex, and supplementary motor cortex. Minor differences existed between two acupoints.

Compared with the resting state prior to acupuncture, brain effects about 10 minutes after needle removal showed significant changes in local functional connectivity and slight changes in distant functional connectivity (Figure 7, Table 1).

Post-EA effects were derived by comparing the degree connectivity of post-EA session with the resting state session. Enhanced local functional connectivity was observed in medial frontal cortex, orbital gyrus, anteroinferior portion of the anterior cingulate cortex, hippocampus, parahippocampus, substantia nigra in the mid-brain, and superolateral gyrus of the frontal lobe (from strong to weak) during the post-EA period for both acupoints. Local connectivity slightly increased in amygdala for CV12, and in hypothalamus for CV4. Decreased local functional connectivity was found in primary somatosensory cortex, visual cortex, and supplementary motor cortex.

In addition, slight differences were found in the local connectivity maps between these two acupoints. Post-EA local connectivity at the somatosensory center was weaker for CV12 as compared to CV4.

## 4. Discussion

The specificity of acupoints is an important issue in acupuncture research and clinical practice. Some fMRI studies of acupuncture showed that visual cortex, auditory cortex, and language cortex were activated during acupuncture at acupoints with regulatory effects on visual, auditory, and language disorders [6, 16–19]. Therefore, some researchers believed that a specific relationship may exist between acupoints and functional regions of the human brain. However, further studies have demonstrated that acupoints are more likely to share similar central effects [5, 7, 8, 11–15, 22, 25, 26, 29]. In addition, Hui et al. [7, 8, 15, 22, 25] found that besides minor differences, acupuncture at different acupoints showed very similar modulatory effects in the limbic system (deactivation effects). These findings were supported by studies with different acupoints using either manual acupuncture or electroacupuncture [11, 25, 26, 29]. In addition, our previous study found that acupuncture had salient modulatory effects on the limbic-paralimbic-neocortical network (LPNN) [12]. The results of the present study provided further evidence to support these previous findings.

Acupoints CV4 and CV12 locate on the middle line of the abdomen, and both belong to the “conception vessel” (CV, Ren Mai). The anatomical structure of these two acupoints predominantly consists of the connective tissues. However, these two acupoints have different nerve innervations (CV4 is innervated by T12 while CV12 by T7/8). According to classic acupuncture theories and clinical reports, CV4 has invigorating effects on the whole body and can regulate the function of the genitourinary system, whereas CV12 has regulatory effects on functions of the gastrointestinal system. The similarity and difference between these two acupoints deserve more detailed investigation.

### 4.1. Similar Needling Sensations between CV4 and CV12

Deqi sensations like soreness, numbness, fullness, and heaviness are the key factors in the therapeutic effectiveness of acupuncture treatment [39–41]. In a study of manual acupuncture at LI4, ST36, and LV3 in 42 healthy volunteers, Hui et al. [38] found that the needling sensations were aching, soreness, pressure, tingling, and numbness with a descending order of prevalence and intensity, whereas in the present study, we found that fullness and numbness were more prominent than soreness and pressure. The differences in sensational prevalence and intensity may be caused by: (1) acupoints used in the study by Hui et al. [38] are in the muscle layer on limbs, where muscle spindles can transmit soreness [42] whereas in our study, acupoints are in the connective tissue layer which may coil around needles easily and cause fullness sensation [42, 43]; (2) differences between manual acupuncture and electroacupuncture.

Furthermore, significantly higher intensity of fullness was found in CV4 than CV12, while manipulation techniques, electric current, meridian category, and tissue structures were the same for both acupoints. We suspect the significant difference in intensity of fullness at these two acupoints may be caused by different sensory receptors in different nerve innervations (CV4 by T12, and CV12 by T7/8). Other potential causes include individuality, small sample size.

### 4.2. Similar Deactivation Effects in the Medial Prefrontal Lobe between EA at CV4 and CV12

EA at both CV4 and CV12 showed significant deactivation effects in the anterior cingulate cortex and the inferior medial prefrontal cortex, and both regions are closely related to pain emotion processing, attention, and the autonomous central nervous system [34, 44–47]. Through comparisons of central effects of 2 Hz and 100 Hz EA and manual acupuncture at ST36, Napadow et al. [11] found similar deactivation effects in medial frontal lobe, anterior cingulate cortex, hippocampus, and amygdala. The results of the present study were partially consistent with their findings. However, we did not find apparent deactivation effects in hippocampus and amygdala. This variation could be due to the differences in the electric stimulatory frequencies and the selection of acupoints.

 Similar to the results reported in the EA study by Napadow et al. [11] and a transcutaneous electric stimulation study by Jin et al. [48], no deactivation effect was found in the precuneal gyrus (medial parietal lobe BA7, BA31). However, the precuneal gyrus showed strong and broad deactivation effect in some manual acupuncture studies [5, 7–9, 12, 29]. This result may indicate the predominant differential brain effects between EA and manual acupuncture. This hypothesis is subject to further investigation.

### 4.3. Sham EA Elicits Moderate Changes of Local FC in the Brain

Sham EA was employed in our design to test whether needle retention was causing the main effects of EA. Sham EA only induced moderate changes in local connectivity in regions apart from the areas showing main EA effects. Moderate enhancement of local connectivity was observed in the medial frontal cortex and anterior cingulate cortex.

### 4.4. EA Modulating Some Core Regions Included in the Default Network

The default network plays an important role in attention, memory, consciousness, and self-referential processes [34, 49]. It includes the inferior parietal lobule (IPL) (BA 40, and 39), the posterior cingulate (BA 30, 23, and 31) and precuneus (BA 7), areas of the inferior, medial and superior frontal gyri (BA 8, 9, 10, and 47), the hippocampal formation, and the lateral temporal cortex (BA 21) [49]. Interesting, it partially overlaps with the limbic-paralimbic-neocortical network, where we observed salient effects of acupuncture in previous study [12]. However, the latter includes more limbic-paralimbic structures, such as amygdala, parahippocampus, hypothalamus, temporal pole, and subgenual anterior cingulate cortex [12, 13, 15]. In the present study, we found that EA at CV4 or CV12 strongly enhanced the local functional connectivity in some regions that belong to the default network and the limbic-paralimbic-neocortical network.

Compared to the sham condition, EA significantly enhanced the local connectivity in medial prefrontal lobe and anterior cingulate cortex, while slight enhancement was observed in hippocampus, parahippocampus, temporal pole, medial parietal lobe, reticular formation of the brain stem, and vermis in the cerebellum. These structures have limbic or paralimbic functions. Hui et al. [15] applied independent component analysis (ICA) to MRI and found that the manual acupuncture at LI4, ST36, and LV3 enhanced the functional connectivity in similar structures as shown in our present study which include the subgenual cingulate, medial prefrontal cortex, and temporal lobe. However, functional connectivity in the medial parietal cortex (precuneal gyrus BA7, BA32) was strongly enhanced by manual acupuncture in Hui's study, which was not observed in the present EA study. The difference may imply different effects on local connectivity in the precuneal gyrus between manual and Electro acupuncture.

### 4.5. Postacupuncture Effects Characterized by the Enhancement of Limbic-Paralimbic-Neocortical Functional Connectivity

Neuroscience studies and clinical trials have found that acupuncture has instant and delayed effects in the management of pain and various disorders [2–4]. However, studies on delayed effects of acupuncture require long-term observation and may be biased by various confounding factors during this process. The emergence of resting state fcMRI technology makes instant observation of delayed effects of acupuncture feasible.

The first investigation of the sustained effects of acupuncture was conducted on resting state connectivity by Dhond et al. [13]. the Probabilistic independent component analysis was used to separate resting fMRI data into DMN and SMN components, and their results demonstrated that acupuncture could enhance the poststimulation spatial extent of resting brain networks to include antinociceptive, memory, and affective brain regions. Bai et al. [14] used a “seeding” method to explore the sustained modulation effects of manual acupuncture and found that acupuncture could cause prominent and long-lasting modulation effects on the intrinsic coherences of the wide interceptive-autonomic areas. In addition, our previous studies have demonstrated that manual acupuncture could modulate the limbic-paralimbic-neocortical network [12, 15]. In the present study, we found that limbic-paralimbic-neocortex functional connectivity during and after EA were both enhanced. An interesting observation was that the post-EA effect resembled the EA effect for both acupoints. However, the post-EA effect seemed more extensive and stronger than in EA, especially in the sensorymotor system. Therefore, we hypothesize that EA may not only have instant modulation effects, but also long-term regulation effects on the brain network.

In the present study, salient post-EA effects at CV4 and CV12 were characterized by enhanced local functional connectivity in medial prefrontal cortex, orbital gyrus, anteroinferior portion of the anterior cingulate cortex, hippocampus, amygdale, and hypothalamus. All of these anatomical structures can be categorized into the limbic-prefrontal network.

Dhond et al. [13] employed manual acupuncture at PC6 and found acupuncture there was increased DMN connectivity with pain (anterior cingulate cortex (ACC) and periaqueductal gray), affective (amygdale and ACC), and memory (hippocampal formation and middle temporal gyrus) related brain regions. These limbic or paralimbic structures are similar with our results.

 In a study of manual acupuncture at ST36, Bai et al. [14] found postacupuncture enhancement of functional connectivity in anterior insular (seed region), dorsolateral frontal cortex, supplementary motor cortex, posterior parietal cortex, and secondary somatosensory area. These areas partially overlap with the areas showing post-EA enhancement of local connectivity in the present study.

Researchers have found that the regulatory function of the emotional circuit in the anterior cingulate cortex was reduced in patients with depression, whereas antidepression medication Venlafaxine could enhance the regulatory effects of the cingulate cortex [50, 51]. Interestingly, as shown above, our results indicated that the major part of this emotional circuit can be modulated by acupuncture. Therefore, we hypothesize that the therapeutic effectiveness of acupuncture on some psychological disorders and pain may depend on the modulation of LPNN by acupuncture.

With a study for CV4 paired with CV12, we aimed to rule out the influences of different anatomical structures, meridians, locations, and order of study procedures. Therefore, the single differentiating factor was the nerve innervation of these two acupoints. Electroacupuncture (EA) has the advantage of standardized stimulation parameters including the frequency and intensity. Therefore, the results are more objective compared to the manual acupuncture. Nonetheless, this present study did not exclude the possible differences between two acupuncturists' manipulations although similar manual acupuncture techniques were applied in the study. In addition, this nonrandomized study was carried out in a single institution on only 21 healthy young volunteers at two abdominal acupoints; therefore, the results of this study may not well characterize the response of the general population, patients with certain specific diseases, or general acupoints. For a better understanding of the needling sensation and the central effects of EA at different acupoints, and a better understanding of the different effects between manual acupuncture and EA, further randomized control trials utilizing EA and manual acupuncture at different acupoints on a large sample of healthy volunteers and patients at different medical centers are warranted.

## 5. Conclusion

EA at CV4 and CV12 produced similar needling sensations. EA elicited salient modulatory effects on limbic-prefrontal and the default network, and the effects persisted after the stimulation.

## Figures and Tables

**Table 1:** The regions showing post-EA effects.

	Resting state (CV4)—True RS				Resting state (CV12)—True RS					CV4 versus CV12		
Region (BA)	Lt (MNI Coordinate)	*T* value	Rt (MNI)	*T*	Lt (MNI)	*T*	Rt (MNI)	*T*	Lt (MNI)	*T*	Rt (MNI)	*T*
MPF BA10/11	−12, 48, −8	4.8	8, 48, −14	5.9	−4, 44, −14	4.5	6, 48, −10	3.32				
Orbital F BA11/12			16, 48, −12	5.67			18, 48, −12	3.09				
Ventra ACC BA24/32	−6, 44, −4	4.3	4, 40, −10	5.32	−8, 40, 2	3.8	6, 34, −12	4.54				
BA25	−8, 12, −12	2.8	2, 14, −14	3	−6, 18, −18	2.8	4, 10, −14	2.34				
FP BA10					−16, 60, 10	2.1						
Hypothalamus			2, 4, −12	2.76								
Hp, HP	−34, −30, −8	3.5	18, −14, −20	2.01	−40, −28, −18	3.8						
Amygdala							24, −8, −20	2.5			24, −8, −20	2.34
TP							38, 18, −42	3.24				
SN			14, −12, −10	2.14			14, −12, −12	2.49				
PCC BA	−10, −72, 50	1.88										
LMF BA	−38, 24, 40	2.8			−24, 20, 42	2.6						
Marginal gyrus BA40			54, −32, 38	2.67							44, −34, 22	2.6
PAG					−6, −34, −8	2.7			−4, −36, −8	2.46		
Cerebellum					−10, −46, −58	3.7			−8, −76, −35	2.71		

*Note*. (for CV4, *n* = 20, for CV12, *n* = 18, *P* < 0.05/*T* > 1.3) True RS (resting state): The resting state session before sham EA.

MPF: medial prefrontal cortex, Orbital F: orbital frontal cortex, Ventra ACC: ventral anterior cingulate cortex, FP: frontal pole, HP: hippocampus, ParaHP: parahippocampus, TP: temporal pole, SN: substantial nigra, PCC: posterior cingulate cortex, LMF: lateral middle frontal cortex, and PAG: periaqueductal gray matter.

The brain regions showing enhanced post-EA local connectivity were similar for two acupoints: medial prefrontal cortex BA10/11, anteroinferior portion of the anterior cingulate cortex BA24/32, BA25, followed by hippocampus, parahippocampus. Differences were seen in Amygdala and Marginal gyrus, BA40 (CV4 > CV12).
